# Ultra-high dose rate dependent modeling of plasmid DNA damage with TOPAS-nBio

**DOI:** 10.1088/1361-6560/ae62c6

**Published:** 2026-05-06

**Authors:** Thongchai A M Masilela, J Naoki D-Kondo, Wook-Geun Shin, Mohammad Rezaee, Jay A LaVerne, Harald Paganetti, Bruce Faddegon, Jan Schuemann, José Ramos-Méndez

**Affiliations:** 1 Department of Radiation Oncology, University of California San Francisco, San Francisco, CA 94115, United States of America; 2 Department of Radiation Oncology, Massachusetts General Hospital and Harvard Medical School, Boston, MA 02114, United States of America; 3 Department of Radiation Oncology and Molecular Radiation Sciences, Johns Hopkins University School of Medicine, Baltimore, MD, United States of America; 4 Radiation Laboratory and Department of Physics and Astronomy, University of Notre Dame, Notre Dame, IN 46556, United States of America

**Keywords:** TOPAS-nBio, Monte Carlo modeling, FLASH, DNA damage, plasmids

## Abstract

*Objective.* FLASH radiotherapy (FLASH-RT) delivers radiation at ultra-high dose rates (UHDR) and has been shown to spare normal tissue while maintaining tumor control (FLASH effect). This could be due to a reduction in radiation-induced DNA damage in normal tissue. Consequently, plasmid assays have been proposed as a way to evaluate these potential differences. However, experimental results have been varied. Track-structure Monte Carlo (MC) simulations may offer a way to disentangle these differences. In this work, we propose a MC model of plasmid DNA damage at UHDR using TOPAS-nBio. *Approach*. The radiolysis of plasmids (pUC19, 50 *μ*g ml^−1^) in an oxygenated (21%) aqueous solution containing DMSO (0.01–100 mM) were modeled in-silico using TOPAS-nBio. 100 Gy was deposited in the solution by 225 kVp x-rays, delivered in a single pulse at conventional (CONV) dose rates (0.1 Gy s^−1^) or UHDR (2 × 10^7^ Gy s^−1^). Two models were evaluated, model 1 in which there was no DNA repair, and model 2 in which oxygen competition was introduced in the form of WR-1065 to induce chemical repair. These models were compared against published experimental data. *Main results*. At CONV dose rates, the model reproduced published experimental single strand break (SSB) yields across a range of scavenging capacities, with statistical uncertainties within 2% (one standard deviation). At low scavenging capacities, there was a 54.7% reduction in SSBs and a 73.5% reduction in double strand breaks at UHDR compared to CONV. At biologically relevant scavenging capacities this difference was within the statistical uncertainty, and there were no observed differences in chemical repair by WR-1065 between UHDR and CONV. *Significance*. These results suggest that the reduction in DNA damage observed experimentally at low DNA concentrations and low scavenging capacities is due to the intertrack effect, with no difference predicted at low DNA concentrations and cell-like scavenging capacities.

## 1. Introduction

FLASH radiotherapy (FLASH-RT), which uses ultra-high dose rate (UHDR) radiation (typically ⩾ 40 Gy s^−1^), has been shown to spare normal tissues while maintaining tumor control (Favaudon *et al* 2014, Borghini *et al* 2024). The mechanisms underpinning this ‘FLASH effect’ are not fully understood, with proposed hypotheses including radical-radical recombination and intertrack effects, radiolytic oxygen depletion, and immune modulation (Abolfath *et al* 2020, Zhou 2020, Rothwell *et al* 2021, Friedl *et al* 2022, Vozenin *et al* 2022). The increased sparing of normal tissue at UHDR compared to conventional (CONV) dose rates could be due to a decrease in DNA damage (Buonanno *et al* 2019, Fouillade *et al* 2020, Levy *et al* 2020, Tessonnier *et al* 2021, Cooper *et al* 2022). Consequently, DNA plasmids have been proposed and are used as a tool to quantify this differential (Small *et al* 2021, Kacem *et al* 2022, Ohsawa *et al* 2022, Perstin *et al* 2022, Konishi *et al* 2023, Sforza *et al* 2024, Wanstall *et al* 2024, Kunz *et al* 2025, Wang *et al* 2025).

Plasmids are short double-stranded DNA molecules found in bacteria and other microscopic organisms. They have been used as biophysical dosimeters to quantify the effects of ionizing radiation (IR), based on radiation induced changes in their conformation (Hempel and Mildenberger 1987, Milligan *et al* 1993, Chen *et al* 1995). Undamaged plasmids are supercoiled, become circular after a single strand break (SSB), and become linear after a double strand break (DSB). Through agarose gel electrophoresis, these structural changes are quantifiable owing to their different velocities within the gel—resulting in distinct band intensities (McMahon and Currell 2011, Maayah *et al* 2022). Plasmids are useful as they allow the study of pure radiation-chemical differences in DNA damage as a function of dose rate without the additional complexities of a biological system, such as histone proteins or enzymatic repair processes, which would otherwise cloud this effect. A better understanding of these different physico-chemical pathways is an important step towards uncovering the mechanism behind FLASH normal tissue sparing (Wardman 2020).

Plasmids can be irradiated in dry or aqueous environments, at different levels of oxygenation, and at various radical scavenger concentrations, thereby mimicking cellular conditions. However, published experimental results have been mixed. Studies performed over a range of ${} _{\text{ }}^{\bullet }{\mathrm{OH}}$ scavenging capacities report little or no difference in DNA SSBs between UHDR and CONV (Small *et al* 2021, Kacem *et al* 2022, Kunz *et al* 2025) while others report statistically significant differences (Ohsawa *et al* 2022, Perstin *et al* 2022, Konishi *et al* 2023, Wanstall *et al* 2024, Wang *et al* 2025). Consequently, computational models of plasmid DNA damage at UHDR are needed to assist in disentangling these differences.

The creation of these computational models can be achieved through Monte Carlo (MC) simulations such as TOPAS-nBio (Schuemann *et al* 2019), the radiobiological extension of TOPAS (Perl *et al* 2012, Faddegon *et al* 2020). By simulating track structure (TS) physics and subsequent chemistry, TOPAS-nBio can be used to study the radiobiological effects of IR at the cellular and subcellular scale. TOPAS wraps Geant4 (Agostinelli *et al* 2003, Allison *et al* 2006, 2016), and TOPAS-nBio wraps and extends Geant4-DNA (Incerti *et al* 2010). Both tools undergo routine benchmarking and regression testing to maintain consistency and accuracy (Arce *et al* 2021, 2025, Masilela *et al* 2025). TOPAS-nBio has previously been validated for the simulation of water radiolysis of low LET radiation in pure liquid water, modeling of scavenger systems to reproduce experimental $e_{{\mathrm{aq}}}^ - $, ${\mathrm{O}}{{\mathrm{H}}^{\bullet }}$, and ${{\mathrm{H}}^{\bullet }}$ radical yields, long-time chemistry, plasmid DNA damage at various scavenging capacities at CONV dose rates, and ${{\mathrm{H}}_{\mathrm{2}}}{{\mathrm{O}}_{\mathrm{2}}}$ production in pure liquid water and cellular-like media at UHDR (Ramos-Méndez *et al* 2020, 2021, D-Kondo *et al* 2025, Shin *et al* 2025). Together, these studies support the use of TOPAS-nBio as a tool to investigate dose rate effects on plasmid DNA damage in various environments.

There are suggestions that the differential response to UHDR in normal and tumor tissues is a combination of two mechanisms that are related to the formation of DNA damage and its subsequent repair. However, no study conclusively quantifies the degree of influence of both mechanisms or which repair pathways are involved (Labarbe *et al* 2020, Levy *et al* 2020, Zhou 2020, Rosini *et al* 2025). In addition to investigating the purely radiation-chemical differences arising due to the use of UHDR, we also incorporated a ‘competition’ mechanism into the model, which competes with oxygen fixation of DNA damage to mimic *in vivo* enzymatic repair. This competition can be induced in the context of plasmid irradiations by using WR-1065, which is a synthetic thiol that reacts with hydrogen abstracted DNA to chemically repair the molecule (Milligan *et al* 1995, 1997, Dziegielewski *et al* 2010). Past work with TOPAS-nBio has successfully implemented WR-1065 in this context to reproduce experimental hypoxia reduction factor values over a range of oxygen concentrations (D-Kondo *et al* 2024).

This work investigates (1) the UHDR induced differences in plasmid DNA SSBs and DSBs over a range of ${} _{\text{ }}^{\bullet }{\mathrm{OH}}$ radical scavenging capacities owing to purely radiation-chemical phenomena, and (2) the impact of the introduction of an oxygen competition mechanism to mimic *in vivo* enzymatic repair.

## 2. Materials and methods

**Figure 1.**
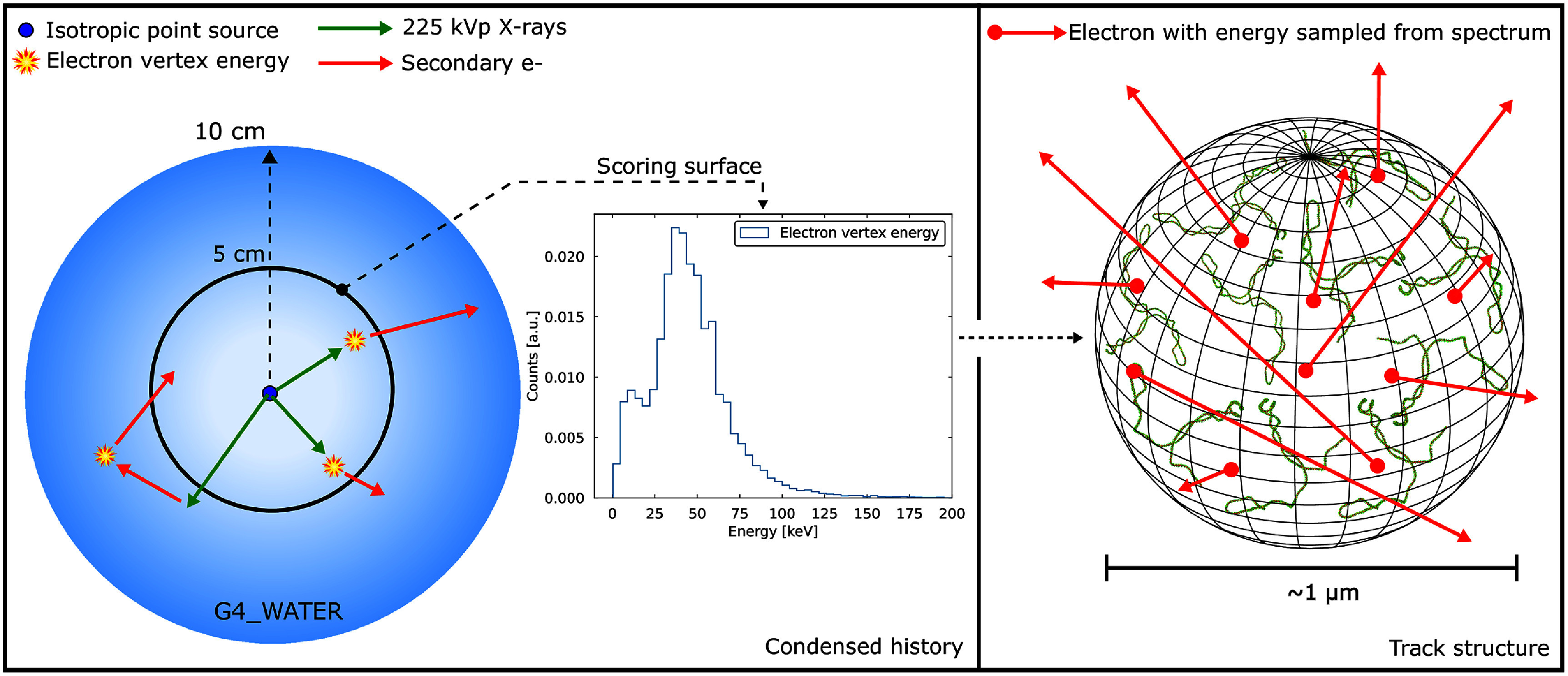
Schematic representation of the TOPAS-nBio simulations, for illustrative purposes only and not drawn to scale. The left panel depicts the condensed history simulations. The blue point is representative of an isotropic point source of 225 kVp x-rays, located at the center of two concentric spheres with radii of 5 cm and 10 cm respectively. The vertex energy of secondary electrons crossing the surface of the 5 cm sphere were scored, resulting in the depicted spectrum. This spectrum was used as the source for the track structure simulations shown in the right panel, where 10 plasmids were placed within a ∼1 *μ*m diameter sphere.

All simulations were performed using TOPAS version OpenTOPAS v4.0.0 (https://opentopas.github.io) and a developer version of TOPAS-nBio v4.0, built on Geant4-11.1.3. The simulation setup is shown in figure 1.

This figure depicts a commonly used approach in multi-scale modeling whereby the simulation is split into two distinct steps. In the first step simulation, condensed history physics is used, which serves as the input to the second step simulation in which chemical processes are activated, and TS physics is employed.

### 2.1. Condensed history simulations

A cut for all particles of 0.05 mm was used in the condensed history simulations. Any particles that would travel less than this distance are not tracked and their energy is locally deposited. In order to ensure conditions of charged particle equilibrium, the simulation setup consisted of an isotropic point source corresponding to the 225 kVp x-ray spectrum of a SARRP (Miles *et al* 2023). As depicted in figure 1, this point source was placed at the center of two concentric spheres of water of 5 and 10 cm radius respectively, and a total of 5 × 10^8^ histories were simulated. A ‘vertex’ in TOPAS-nBio refers to the initial kinetic energy, position, and momentum of a particle after it has been generated through a physical process. The vertex kinetic energy of any liberated secondary electrons crossing the surface of the 5 cm sphere was scored, and only these energies contributed to the electron energy spectrum while all other electrons were ignored. In accordance with a recent recommendation for the accurate simulation of low energy electrons using the condensed history approach, the electromagnetic physics list *G4EMStandardPhysics_opt4* was used (Arce *et al* 2021). Each primary electron simulated in the TS simulations was assigned an energy sampled from the vertex energy spectrum resulting from the condensed history simulations. The same methodology was previously employed in the validation of TOPAS-nBio for the calculation of plasmid strand breaks (Ramos-Méndez *et al* 2021).

### 2.2. Track structure simulations

#### 2.2.1. Track structure source and DNA concentration

As described above, a spectrum of secondary electrons was used as the source in the TS simulations. To model a uniform pulse of radiation, TOPAS-nBio requires the pulse width (FWHM and mean), and a prescribed dose, thereby enabling the modeling of dose rate effects by modifying either of these input parameters. Each particle within the pulse was assigned a time sampled from the uniform distribution, and an initial energy sampled from the spectrum of secondary electrons. This particle time information was inherited by all secondaries produced by the primary particle, and all chemical species created by the end of the physico-chemical stage. Before activating any chemistry processes, the physical track of each history was simulated in its entirety. The dose deposited by each history was accumulated until the prescribed dose within the microscopic volume of interest was reached, following which the chemistry of the simulation was activated, and the independent reaction times (IRT) algorithm was run (Ramos-Méndez *et al* 2020). We assume that there is no qualitative difference in the radiation chemistry occurring within microscopic volumes compared to macroscopic (∼cm) volumes provided that the dose and dose rate remain the same. For all simulations described in this work a volumetric source was used, with initial electron positions uniformly distributed within the plasmid containing volume, and each electron assigned an isotropic initial direction. By placing the electrons uniformly with isotropic directions, we approximate the local radiation field produced by secondary electrons, independent of the macroscopic irradiation geometry. A dose of 100 Gy was delivered at a dose rate of 0.1 Gy s^−1^ (1000 s FWHM) for CONV and 2 × 10^7^ Gy s^−1^ (5 *μ*s FWHM) for UHDR. Single pulse irradiations at this dose rate have been shown to induce the FLASH effect (Schulte *et al* 2023). To model a specific DNA concentration, equation (1) was used. The spherical radius of the water volume is represented by *r*. This volume contains *N* pUC19 plasmids, with each plasmid containing 2686 base pairs with a molar mass *M* of 660 g mol^−1^ for each base pair. The concentration of plasmids within the volume is *C*, and *N*_A_ is Avogadro’s constant,

\begin{equation*}r = { }\sqrt[3]{{\frac{3}{{4\pi }} \times \frac{{N \times M \times {\mathrm{bp}}}}{{C \times {N_{\mathrm{A}}}}}}}.\end{equation*}

Assuming a 5 *μ*g ml^−1^ concentration, the spherical radius required for a single plasmid within this volume of water corresponds to 0.52 *μ*m. Calculating the radius in this way is useful computationally as the only parameter that now needs to be changed to modify the concentration simulated is the number of plasmids placed within this volume. For all simulations in this work, 10 pUC19 plasmids were placed with random positions and orientations in the sphere. To avoid overlaps, each plasmid’s position and orientation was resampled if it failed Geant4’s *G4VPhysicalVolume::CheckOverlaps* method (D-Kondo *et al* 2021). The 10 plasmids used in this work corresponds to a DNA concentration of 50 *μ*g ml^−1^. A sensitivity analysis was performed by increasing the concentration of DNA simulated by a factor of 5, and evaluating the impact of changing the DSB induction restriction from 10 bp to 5 and 15 bp. This was done to evaluate potential systematic uncertainties in the distribution of DSBs, linked to the low DNA concentration of a dissolved plasmid solution.

#### 2.2.2. Physical and chemical processes

The process of water radiolysis can be broken down into three relatively distinct stages, namely the physical, physico-chemical, and non-homogeneous chemical stages which occur on timescales of roughly < 1 fs, 1 fs to 1 ps, and 1 ps to 1 *μ*s respectively. For the physical stage, the customizable TOPAS-nBio physics list *TsEmDNAPhysics* was used, which is based on the ‘option 2’ physics constructor of Geant4-DNA, as described elsewhere (Incerti *et al* 2018). Taking advantage of its customizability, the elastic scattering model used in *TsEmDNAPhysics* was changed from the Champion model (Champion *et al* 2009) to the ELSEPA model (Salvat *et al* 2005), which was shown to achieve better agreement with experimental *G* values (Shin *et al* 2019). The thermalization of subexcitation electrons into aqueous electrons in the physico-chemical stage was handled by the Meesungnoen Geant4-DNA model (Meesungnoen *et al* 2002, Shin *et al* 2019).

The reaction kinetics of the non-homogeneous chemical stage and beyond was performed using the IRT algorithm (Clifford *et al* 1986, Green *et al* 1990, Pimblott *et al* 1991), which was previously validated and implemented into TOPAS-nBio (Ramos-Méndez *et al* 2020, 2021). The IRT works by first generating a reaction time for each pair of species present in a system, sorting those times in ascending order, then performing the reaction with the shortest time. The reactant pair and all other reactions involving that pair are removed. If products are generated, reaction times are sampled between the species already present in the system and the new species, reaction times are added to the sorted list, and the algorithm then performs the next reaction with the shortest reaction time (Plante 2021). The modeling of first order and background reactions was performed using the exponential function shown in equation (2) where *k*_obs_ is the observed reaction rate (/M s^−1^), *B* is the concentration (*M*) of solute species assumed to be uniformly distributed in the medium, *t* is the time (s), and *P* is the probability of reaction,

\begin{equation*}P = 1 - {{\mathrm{e}}^{ - {k_{{\mathrm{obs}}}}Bt}}.\end{equation*}

The full set of chemical reactions used is displayed in table 1. For instances where the product of the reaction is listed as ‘product’, it is assumed that the created species has no bearing on the rest of the system. These aspects are expanded upon in the discussion. Reactions with and without an asterisk refer to first- and second-order reactions respectively. The observed reaction rates, ${k_{{\mathrm{obs}}}}$, were provided for all second-order reactions in units of /M s^−1^, whereas for first-order reactions ${k_{{\mathrm{obs}}}}$ needs to be multiplied by the concentration of the homogeneously distributed solute species. This modification gives a scavenging capacity in units of s^–1^, to be used in equation (2). The molar concentration of 10^−7^ M used for reactions R3* and R11* corresponds to the concentration of hydroxide (${\mathrm{O}}{{\mathrm{H}}^ - }$) and hydronium (${{\mathrm{H}}_{\mathrm{3}}}{{\mathrm{O}}^ + }$) ions at a neutral pH and 25 °C. Diffusion coefficients of each molecular species were taken from (Plante and Devroye 2017). Default reactions and oxygen reactions were taken from (Pimblott 1992), with additional pure liquid water reactions taken from (Pastina and LaVerne 2001, Plante 2021) in line with previous work (D-Kondo *et al* 2021). Unless otherwise stated, all reaction rates were taken from the Buxton compilation (Buxton *et al* 1988).

**Table 1.** Full list of chemical reactions and associated reaction rates employed in the TOPAS-nBio models. Reactions marked with an asterisk were modeled as first order reactions according to equation (2). For instances where the product of the reaction is listed as ‘product’, it is assumed that the created species has no bearing on the rest of the system.

1. Default TOPAS-nBio pure liquid water reactions					

No.	Reaction	${k_{{\mathrm{obs}}}}$ [/M s^−1^ or /s]	No.	Reaction	${k_{{\mathrm{obs}}}}$ [/M s^−1^ or /s]

R1	$e_{{\mathrm{aq}}}^ - + e_{{\mathrm{aq}}}^ - \to 2{\mathrm{O}}{{\mathrm{H}}^ - } + {{\mathrm{H}}_2}$	$1.1 \times {10^{10}}$	R7	${{\mathrm{H}}^{\bullet }}{\text{ + }}{{\mathrm{H}}^{\bullet }} \to {{\mathrm{H}}_{\mathrm{2}}}$	$1.56 \times {10^{10}}$
R2	$e_{\mathrm{aq}}^ - + {{\mathrm{H}}_{\mathrm{3}}}{{\mathrm{O}}^ + } \to {{\mathrm{H}}^ \bullet }$	$2.3 \times {10^{10}}$	R8	${{\mathrm{H}}^{\bullet }}{\text{ + }}{}_{\text{ }}^{\bullet }{\mathrm{OH}} \to {{\mathrm{H}}_{\mathrm{2}}}{\mathrm{O}}$	$2.0 \times {10^{10}}$
R3*	$e_{\mathrm{aq}}^ - + {{\mathrm{H}}_{\mathrm{3}}}{{\mathrm{O}}^ + } \to {{\mathrm{H}}^ \bullet }$	$2.3 \times {10^{10}} \cdot \left[ {{{10}^{ - 7}}} \right]$	R9	${{\mathrm{H}}^\bullet} + {{\mathrm{H}}_2}{{\mathrm{O}}_2}{ \to ^\bullet}{\mathrm{OH}} + {{\mathrm{H}}_2}{\mathrm{O}}$	$9.0 \times {10^7}$
R4	$e_{\mathrm{aq}}^ - + {{\mathrm{H}}^ \bullet } \to {{\mathrm{H}}_{\mathrm{2}}}{\text{ + O}}{{\mathrm{H}}^ - }$	$2.5 \times {10^{10}}$	R10	${{\mathrm{H}}_{\mathrm{3}}}{{\mathrm{O}}^{\text{ + }}}{\text{ + O}}{{\mathrm{H}}^ - } \to {{\mathrm{H}}_{\mathrm{2}}}{\mathrm{O}}$	$14.3 \times {10^{10}}$
R5	$e_{\mathrm{aq}}^ - { + ^\bullet}{\mathrm{OH}} \to {\mathrm{O}}{{\mathrm{H}}^ - }$	$3.0 \times {10^{10}}$	R11*	${{\mathrm{H}}_{\mathrm{3}}}{{\mathrm{O}}^ + } + {\mathrm{O}}{{\mathrm{H}}^ - } \to {{\mathrm{H}}_{\mathrm{2}}}{\mathrm{O}}$	$14.3 \times {10^{10}} \cdot \left[ {{{10}^{ - 7}}} \right]$
R6	$e_{\mathrm{aq}}^ - + {{\mathrm{H}}_2}{{\mathrm{O}}_2} \to {\mathrm{O}}{{\mathrm{H}}^ - }{ + ^\bullet}{\mathrm{OH}}$	$1.1 \times {10^{10}}$	R12	$^\bullet{\mathrm{OH}}{ + ^\bullet}{\mathrm{OH}} \to {{\mathrm{H}}_2}{{\mathrm{O}}_2}$	$1.1 \times {10^{10}}$

2. Additional pure liquid water reactions for homogeneous regime					

R13	$e_{\mathrm{aq}}^ - + {\mathrm{HO}}_{\mathrm{2}}^{\bullet } \to {{\mathrm{H}}_{\mathrm{2}}}{{\mathrm{O}}_{\mathrm{2}}}{\text{ + O}}{{\mathrm{H}}^ - }$	$2.0 \times {10^{10}}$	R20	${\mathrm{HO}}_{\mathrm{2}}^{\bullet } + {\mathrm{O}}_2^{ \bullet - } \to {{\mathrm{H}}_{\mathrm{2}}}{{\mathrm{O}}_{\mathrm{2}}}{\text{ + }}{{\mathrm{O}}_{\mathrm{2}}}{\text{ + O}}{{\mathrm{H}}^ - }$	$9.7 \times {10^7}$
R14	$e_{\mathrm{aq}}^ - + {\mathrm{O}}_2^{ \bullet - } \to {{\mathrm{H}}_{\mathrm{2}}}{{\mathrm{O}}_{\mathrm{2}}} + 2{\mathrm{O}}{{\mathrm{H}}^ - }$	$1.3 \times {10^{10}}$	R21	${{\mathrm{H}}_{\mathrm{2}}}{{\mathrm{O}}_{\mathrm{2}}}{\text{ + HO}}_{\mathrm{2}}^{\bullet } \to {{\mathrm{O}}_{\mathrm{2}}}{\text{ + }}{}_{\text{ }}^{\bullet }{\mathrm{OH}}$	$5.0 \times {10^{ - 1}}$
R15	${{\mathrm{H}}^{\bullet }}{\text{ + HO}}_{\mathrm{2}}^{\bullet } \to {{\mathrm{H}}_{\mathrm{2}}}{{\mathrm{O}}_{\mathrm{2}}}$	$1.5 \times {10^{10}}$	R22	${{\mathrm{H}}_{\mathrm{2}}}{{\mathrm{O}}_{\mathrm{2}}}{\text{ + O}}_2^{ \bullet - } \to {{\mathrm{O}}_{\mathrm{2}}}{\text{ + }}{}_{\text{ }}^{\bullet }{\text{OH + O}}{{\mathrm{H}}^ - }$	$1.3 \times {10^{ - 1}}$
R16	${{\mathrm{H}}^{\bullet }}{\text{ + O}}_2^{ \bullet - } \to {{\mathrm{H}}_{\mathrm{2}}}{{\mathrm{O}}_{\mathrm{2}}}{\text{ + O}}{{\mathrm{H}}^ - }$	$1.8 \times {10^{10}}$	R23	${H_2}{O_2}{ + ^\bullet}OH \to O_2^{\bullet - } + {H_3}{O^ + }$	$2.7 \times {10^7}$
R17	${{\mathrm{H}}_{\mathrm{3}}}{{\mathrm{O}}^ + } + {\mathrm{O}}_2^{ \bullet - } \to {\mathrm{HO}}_{\mathrm{2}}^{\bullet }$	$5.0 \times {10^{10}}$	R24	${\mathrm{O}}_2^{\bullet - }{ + ^\bullet}{\mathrm{OH}} \to {{\mathrm{O}}_2} + {\mathrm{O}}{{\mathrm{H}}^ - }$	$8.2 \times {10^9}$
R18	${\mathrm{HO}}_{\mathrm{2}}^{\bullet }{\text{ + HO}}_2^ \bullet \to {{\mathrm{H}}_{\mathrm{2}}}{{\mathrm{O}}_{\mathrm{2}}}{\text{ + }}{{\mathrm{O}}_{\mathrm{2}}}$	$8.3 \times {10^5}$	R25	${\mathrm{HO}}_{\mathrm{2}}^{\bullet }{\text{ + }}{}_{\text{ }}^{\bullet }{\mathrm{OH}} \to {{\mathrm{O}}_{\mathrm{2}}}{\text{ }}$	$6.0 \times {10^9}$
R19*	${\mathrm{HO}}_2^ \bullet \to {{\mathrm{H}}_{\mathrm{3}}}{{\mathrm{O}}^{\text{ + }}}{\text{ + O}}_2^{ \bullet - }$	$8.05 \times {10^5}$	R26	$^\bullet{\mathrm{OH}} + {{\mathrm{H}}_2} \to {{\mathrm{H}}^\bullet}$	$4.3 \times {10^7}$

3. Radical reactions with molecular oxygen					

R27	$e_{\mathrm{aq}}^ - + {{\mathrm{O}}_2} \to {\mathrm{O}}_2^{ \bullet - }$	$1.9 \times {10^{10}}$	R29*	$e_{\mathrm{aq}}^ - + {{\mathrm{O}}_2} \to {\mathrm{O}}_2^{ \bullet - }$	$1.9 \times {10^{10}} \cdot \left[ {{{\mathrm{O}}_{\mathrm{2}}}} \right]$
R28	${{\mathrm{H}}^{\bullet }}{\text{ + }}{{\mathrm{O}}_{\mathrm{2}}} \to {\mathrm{HO}}_2^ \bullet $	$2.1 \times {10^{10}}$	R30*	${{\mathrm{H}}^{\bullet }}{\text{ + }}{{\mathrm{O}}_2} \to {\mathrm{HO}}_2^ \bullet $	$2.1 \times {10^{10}} \cdot \left[ {{O_2}} \right]$

4. Radical scavenger reactions					

R31*	$^\bullet{\mathrm{OH}} + {\mathrm{DMSO}} \to {\mathrm{product}}$	$7.1 \times {10^9} \cdot \left[ {{\mathrm{DMSO}}} \right]$	R33*	$e_{\mathrm{aq}}^ - + {\mathrm{DMSO}} \to {\mathrm{product}}$	$3.8 \times {10^6} \cdot \left[ {{\mathrm{DMSO}}} \right]$
R32*	${{\mathrm{H}}^ \bullet } + {\mathrm{DMSO}} \to {\mathrm{product}}$	$2.7 \times {10^7} \cdot \left[ {{\mathrm{DMSO}}} \right]$			

5. Reactions for DNA damage induction and chemical repair					

Model (1)					

R34	${} _{\text{ }}^{\bullet }{\mathrm{OH}} + {\mathrm{DNA}} \to {\mathrm{Break}}$	*See Equation* (4)	R36	$e_{\mathrm{aq}}^ - + {\mathrm{DNA}} \to {\mathrm{product}}$	$1.0 \times {10^7}$
R35	${{\mathrm{H}}^ \bullet } + {\mathrm{DNA}} \to {\mathrm{Break}}$	$2.9 \times {10^7}$			

Model (2)					

R37	$^\bullet{\mathrm{OH}} + {\mathrm{DNA}} \to DN{A^\cdot}$	*See Equation* (4)	R41*	${{\mathrm{O}}_2} + DN{A^ \bullet } \to {\mathrm{Break}}$	$4.0 \times {10^8}$
R38	${{\mathrm{H}}^ \bullet } + {\mathrm{DNA}} \to DN{A^ \bullet }$	$2.9 \times {10^7}$	R42*	$^\bullet{\mathrm{OH}} + WR{\text{ - 1065}} \to {\mathrm{product}}$	$9.2 \times {10^9} \cdot \left[ {WR1065} \right]$
R39	$e_{\mathrm{aq}}^ - + {\mathrm{DNA}} \to {\mathrm{product}}$	$1.0 \times {10^7}$	R43*	$WR{\text{ - }}1065 + DN{A^ \bullet } \to {\mathrm{DNA}}$	$9.4 \times {10^7} \cdot \left[ {WR1065} \right]$
R40	${{\mathrm{O}}_2} + DN{A^ \bullet } \to {\mathrm{Break}}$	$4.0 \times {10^8}$			

For the background reactions with molecular oxygen (R29* and R30*), the concentration of oxygen was determined according to Henry’s law, shown in equation (3), where *C* is the concentration of the dissolved gas (M), *k* is Henry’s law constant (M atm^−1^), and P_O2_ is the partial pressure of oxygen (atm),

\begin{align*}C = k{P_{{\mathrm{O2}}}}.\end{align*}

For atmospheric oxygen of 21% O_2_ and $k = 1.3 \times {10^{ - 5}}$ (Sander 2023), the concentration of oxygen is $C = 1.3 \times {10^{ - 5}} \times 0.21 \times 101325 \approx 0.27 \times {10^{ - 3}}{\text{ M}}$. In order to vary the degree of hydroxyl radical scavenging, dimethyl sulfoxide (DMSO) was used in concentrations of 10^−5^ M, 10^−4^ M, 0.001 M, and 0.1 M, the latter of which results in a level of ${} _{\text{ }}^{\bullet }{\mathrm{OH}}$ radical scavenging the same order of magnitude as that found within a cell (Roots and Okada 1975).

#### 2.2.3. Models

In order to simulate plasmid DNA damage, two different chemical models were used, however the sets of reactions used between UHDR and CONV were the same and none of the parameters in table 1 are dose rate dependent. Possible UHDR induced differences in strand break production in the absence of any repair mechanisms was modeled using the reaction sets 1–4 in table 1, in addition to reactions R34–R36. This model is hereafter referred to as *model 1*. The reaction rate constant of R34 is dependent on the ${} _{\text{ }}^{\bullet }{\mathrm{OH}}$ radical scavenging capacity of the system and can be calculated according to equation (4) (Milligan *et al* 1996, D-Kondo *et al* 2021), where *σ* is the total ${} _{\text{ }}^{\bullet }{\mathrm{OH}}$ radical scavenging capacity of the system,

\begin{equation*}{k_{{\mathrm{obs}}}} = { }1.32 \times {10^7}{\sigma ^{0.29}}.\end{equation*}

The products of R34 and R35 are representative of potential breaks. The actual strand breaks induced by reactions R34 and R35 were obtained by first multiplying the obtained *G* values of these potential breaks with an efficiency term to account for various factors not considered in this work, such as the different hydrogen abstraction sites, reactions with nucleobases, or differences arising due to the specific DNA geometry or underlying MC models. This efficiency factor typically ranges from 12% to 65% (Klimczak *et al* 1993, Milligan *et al* 1993, Udovičić *et al* 1994, Friedland *et al* 2011, D-Kondo *et al* 2021). SSBs calculated in this work were obtained by applying an efficiency of 24% (Ramos-Méndez *et al* 2021) and 0.8% (Aydogan *et al* 2008) to reactions R34 and R35 respectively. These efficiencies were previously obtained using the Nelder–Mead minimization algorithm (Nelder and Mead 1965, Ramos-Méndez *et al* 2021).

In order to calculate the yield of DSBs, after each simulation run the plasmid ID, strand ID, and base ID of all damaged sites was scored and fed into a Python script. These IDs provide positional information corresponding to an interaction of the IR, either direct or indirect, on the plasmid. In order for a DSB to occur, two interactions need to be on the same plasmid, on opposite strands, and within 10 bp of one another. Each interaction is then probabilistically accepted or rejected based on the aforementioned efficiencies using Python’s *random()* function. Consequently, for a DSB to occur both the ID restrictions and efficiency criteria need to be met. This script was run a total of 10^6^ times for each concentration of DMSO and each irradiation modality to obtain a distribution of DSBs.

In the second model, hereafter referred to as *model 2*, oxygen competition in the form of chemical repair by WR-1065 was introduced. Model 2 uses the same dose rates as model 1, reaction sets 1–4 in table 1, and reactions R37–R43*. As shown previously, the inclusion of WR-1065, resulting in a different reaction pathway for the induction of SSBs, necessitates an efficiency of 70% applied to the oxygen fixation reactions R40 and R41* (D-Kondo *et al* 2024). Given the additional ${} _{\text{ }}^{\bullet }{\mathrm{OH}}$ scavenging by WR-1065 (R42*) (Ward and Mora-Arellano 1984), the scavenging capacity σ in equation (4) was recalculated by assuming an additive effect then applied to R37. The reaction rates for R40, R41*, and R43* were taken from Milligan *et al* (Milligan *et al* 1995). The full set of chemistry parameter files for both models will be released as an example in a future version of TOPAS-nBio.

### 2.3. Experimental data selection criteria

To compare the full set of UHDR-irradiated plasmid experiments with the TOPAS-nBio simulations, the percent reduction in strand breaks for CONV vs UHDR was calculated for each experimental study. Although the individual studies differed substantially in particle type (LET), pulse structures, doses, and dose rates, they all reported strand break induction under both CONV and UHDR conditions, and at specific ${} _{\text{ }}^{\bullet }{\mathrm{OH}}$ radical scavenging capacities. This commonality facilitated both the combination of the datasets and plotting the percent reduction in strand breaks as a function of scavenging capacity. In all cases, either Tris (Small *et al* 2021, Ohsawa *et al* 2022, Perstin *et al* 2022, Konishi *et al* 2023, Sforza *et al* 2024, Wanstall *et al* 2024, Wang *et al* 2025) or DMSO (Kunz *et al* 2025) was used as the radical scavenger. The scavenging capacities were obtained by multiplying the concentration of scavenger by the reaction rate constant of its reaction with the ${} _{\text{ }}^{\bullet }{\mathrm{OH}}$ radical. The rate constants of 1.1 $ \times $ 10^9^ M s^−1^ (Hicks and Gebicki 1986) and 7.1 $ \times $ 10^9^ M s^−1^ (Milligan *et al* 1996) were used for Tris and DMSO respectively. In some cases, the stock plasmid solution contains residual EDTA with a pH of ∼7.4 depending on the supplier. Scavenging of the ${} _{\text{ }}^{\bullet }{\mathrm{OH}}$ radical by EDTA, which varies as a function of pH, was also accounted for in the final calculation. A rate constant of 1.49 $ \times $ 10^9^ M s^−1^ was used, which was obtained by interpolation at the aforementioned pH (Höbel and Sonntag 1998). Where possible, the percent reduction in DNA damage from CONV to UHDR was calculated from the raw data points in each study, which themselves were obtained directly from the band intensities from gel electrophoresis. These data points correspond to the plasmid fraction in either open circular (SSB) or linear (DSB) form. This evaluation was performed at the highest dose delivered as most of the differences between UHDR and CONV in plasmid irradiations have been observed at high doses. In some cases, the plasmid fractions for each form were not provided, or if provided, the data points for UHDR and CONV were not at the same delivered dose. In these cases, the ratio was calculated from the reported induction rate of SSBs/DSBs obtained from the model used in the study.

For the experimental studies from which the percent reduction was calculated using the raw plasmid fractions, the dose, particle type, and UHDR instantaneous dose rate (IDR) or mean (MDR) dose rate was: kV x-rays depositing 100 Gy at an MDR of 125 Gy s^−1^ (Sforza *et al* 2024); 16 MeV electrons depositing 30 Gy at an IDR of 1.04 $ \times $ 10^5^ Gy s^−1^ (Perstin *et al* 2022), 6 MeV electrons depositing ∼60 Gy at an IDR of 10^6^ Gy s^−1^ (Wang *et al* 2025); and protons depositing ∼50 Gy at an MDR of 1400 Gy s^−1^ (Kunz *et al* 2025), and ∼63 Gy at 48.6 Gy s^−1^ (Konishi *et al* 2023). For studies in which the induction rate was used, particle types and dose rates were: 201 MeV electrons at an IDR of 2 $ \times $ 10^9^ Gy s^−1^ (Wanstall *et al* 2024) and 200 MeV electrons at an IDR $ &gt; $ 10^8^ Gy s^−1^ (Small *et al* 2021); and protons at 40 Gy s^−1^ (Ohsawa *et al* 2022). Furthermore, two studies made use of Fpg (Konishi *et al* 2023) or Fpg and Nth (Sforza *et al* 2024), which are enzymes that convert base damages into strand breaks. 225 kVp x-rays were simulated for comparison with these experiments given that TOPAS-nBio has been validated for low LET radiation (Ramos-Méndez *et al* 2021), and the particle energies used in these experiments are sufficiently high that no LET effects are expected. The one exception is Kunz *et al*, in which they also irradiated plasmids in the pristine Bragg peak and spread-out Bragg peak (2025). Nevertheless, they observed that while the higher LET increased clustered strand breaks, there was no dose rate dependency, and they saw no significant difference in plasmid damage between UHDR and CONV. Additional information relating to these experimental studies, and figures or tables from which this percent reduction was calculated, is shown in table 2. Values shown in this table refer to the conditions under which the percent reduction in SSBs or DSBs was calculated for inclusion in this work and is not necessarily representative of the full scope of the respective studies.

**Table 2.** Experimental plasmid studies used for the calculation of SSB or DSB percent reduction due to UHDR. Asterisks indicate ratios obtained from SSB induction rates as opposed to raw plasmid fractions.

Study	[DNA] (*μ*g ml^−1^)	^•^OH Scav. Cap. (/s)	Enzyme	Dose (Gy)	Relevant data	SSB Redu. (%)	DSB Redu. (%)
Sforza *et al* (2024)	35	1.10 × 10^6^	yes	100	Taken from figures 3(B) and 4	−22.8	0
		2.75 × 10^8^				0	0
Kunz *et al* (2025)	40	9.94 × 10^7^	no	50	Taken from figure 2(B)	4.2	0
Perstin *et al* (2022)	24	5.28 × 10^5^	no	30	Taken from figures 5(B) and (C)	−10.6 (93.2 Gy s^−1^)	−57.8 (93.2 Gy s^−1^)
						−7.4 (46.6 Gy s^−1^)	−60 (46.6 Gy s^−1^)
Konishi *et al* (2023)	50	1.25 × 10^6^	no	63	Taken from figures 1(B) and (C)	−20.6	0
			yes			−11.3	0
Wang *et al* (2025)	15	3.30 × 10^5^	no	60	Taken from figure 1	−6.4	0
Ohsawa *et al* (2022)*	50	1.25 × 10^6^	no	0–100	Taken from table 1	−19	0
Small *et al* (2021)*	100	1.25 × 10^6^	no	0–50	Taken from table 3	5.1	0
Wanstall *et al* (2024)*	100	1.10 × 10^7^	no	0–200	Taken from table 2	−26.6	0
		1.10 × 10^8^				−16.2	0

## 3. Results

### 3.1. SSB

**Figure 2.**
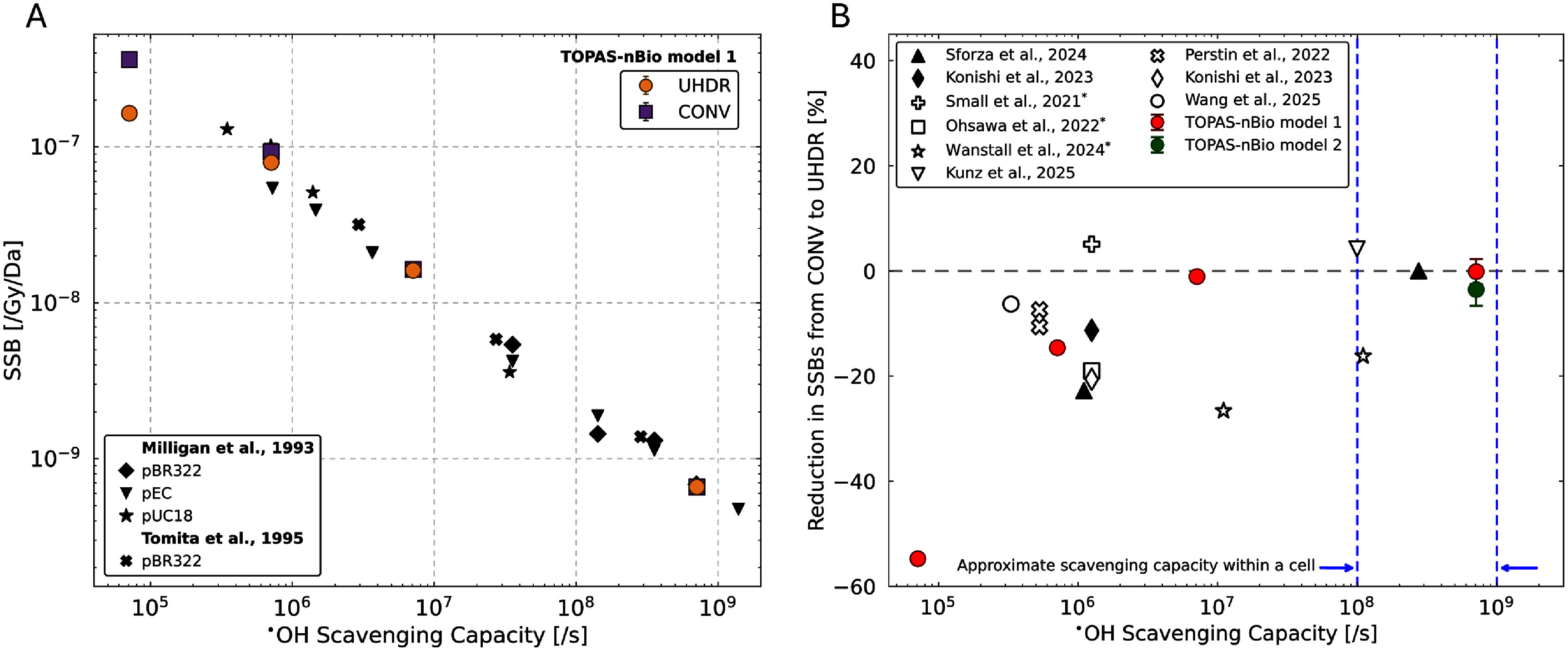
Computed SSBs for TOPAS-nBio model 1 compared to CONV experimental data (Milligan *et al* 1993, Tomita *et al* 1995) as a function of scavenging capacity (A), and percent reduction in SSBs from CONV to UHDR as a function of scavenging capacity for models 1 and 2 (B). For the experimental data of panel (B): empty symbols represent ratios obtained from open circular fractions (Perstin *et al* 2022, Konishi *et al* 2023, Kunz *et al* 2025, Wang *et al* 2025) with the Perstin *et al* datapoints corresponding to a MDR of 46.6 Gy s^−1^ (top) and 93.2 Gy s^−1^ (bottom), filled in symbols indicate that an enzyme (Fpg or Fpg and Nth) was used to convert base damages to strand breaks (Konishi *et al* 2023, Sforza *et al* 2024), and asterisks indicate ratios obtained from SSB induction rates calculated from a model (Small *et al* 2021, Ohsawa *et al* 2022, Wanstall *et al* 2024).

Panel A of figure 2 depicts the variation of SSBs as a function of the radical scavenging capacity. Simulations of model 1 at CONV resulted in a good agreement with the low dose rate experimental data (Milligan *et al* 1993, Tomita *et al* 1995) across a range of scavenging capacities, as shown previously for low LET radiation (Ramos-Méndez *et al* 2021). Increasing the dose rate to UHDR, we observe a reduction in the number of strand breaks at the lower scavenging capacities (<10^7^ s^−1^). Simulations were run until the statistical uncertainty of SSB G values in all conditions were less than 2%. CONV SSBs (/Gy Da^−1^) from the lowest to highest scavenging capacity were (3.63 $ \pm $ 0.01) $ \times $ 10^−7^, (9.31 $ \pm $ 0.05) $ \times $ 10^−8^, (1.63 $ \pm $ 0.01) $ \times $ 10^−8^, and (6.59 $ \pm $ 0.10) $ \times $ 10^−10^, while for UHDR (/Gy Da^−1^) these values were (1.64 $ \pm $ 0.01) $ \times $ 10^−7^, (7.95 $ \pm $ 0.05) $ \times $ 10^−8^, (1.62 $ \pm $ 0.01) $ \times $ 10^−8^, and (6.59 $ \pm $ 0.01) $ \times $ 10^−10^. At the highest scavenging capacity of 0.1 M of DMSO, the SSBs at CONV and UHDR were not statistically significantly different.

These SSB values correspond to an approximate 54.7%, 14.6%, 1.1%, and 0.1% reduction when going from CONV to UHDR for DMSO concentrations of 10^−5^ M, 10^−4^ M, 0.001 M, and 0.1 M, respectively. This percent reduction is displayed in panel B of figure 2. As depicted by model 1, the ratio of CONV to UHDR induced damages rises asymptotically to 0 as the ${} _{\text{ }}^{\bullet }{\mathrm{OH}}$ radical scavenging capacity increases. Model 2 (single point in solid green) introduced a different pathway for SSB induction, first requiring oxygen fixation, that competed with chemical repair by WR-1065. This different pathway resulted in SSBs for CONV and UHDR of 1.03 $ \times {\text{ }}$ 10^−9^ Gy Da^−1^ and 9.92 $ \times $ 10^−10^ Gy Da^−1^ respectively (3.5% reduction). As shown in figure 2, the SSBs at UHDR for model 2 and model 1 agree within 1 standard deviation statistical uncertainty. An additional set of model 1 simulations were performed at the highest scavenging capacity with a dose rate two orders of magnitude higher (2 $ \times $ 10^9^ Gy s^−1^). This was done to match the highest dose rate used in the plasmid experiments (Wanstall *et al* 2024), and to be more in line with the work of Berry *et al* who also observed a FLASH effect using a single pulse dose rate of this magnitude (Berry *et al* 1969, Bourhis *et al* 2019). These results are not shown in figure 2 to maintain visual clarity, however, a SSB yield of (6.48 $ \pm $ 0.23) $ \times $ 10^−10^ /Gy Da^−1^ with a 3.5% uncertainty was obtained, in agreement (1 standard deviation) with the lower dose rate model 1 simulations at the highest scavenging capacity. To evaluate the impact of the range cut, additional TS simulations were performed with the spectrum generated from a 0.1 *μ*m range cut at the lowest scavenging capacity. This resulted in SSB yields at UHDR of (1.60 $ \pm $ 0.01) $ \times $ 10^−7^ Gy Da^−1^, differing by 2.5% from the SSB yields of the 0.05 mm cut, and thus demonstrating that this parameter only has a minor impact under the current setup.

Despite all the experimental studies having been performed under different conditions, there appears to be a greater sparing effect as the ${} _{\text{ }}^{\bullet }{\mathrm{OH}}$ radical scavenging capacity decreases. This is due to fewer SSBs at UHDR compared to CONV, a trend reproduced in the model 1 MC simulations. Taking the inverse of the commonly accepted ${} _{\text{ }}^{\bullet }{\mathrm{OH}}$ radical lifetime of 4 ns (Roots and Okada 1975) yields an average scavenging capacity of 2.5 $ \times $ 10^8^ s^−1^. This region of biologically relevant scavenging was represented by a one order of magnitude range between 10^8^ s^−1^ and 10^9^ s^−1^, as shown in panel B of figure 2. We find that under these conditions there was no significant difference between UHDR and CONV induced plasmid DNA damage. As the scavenging capacity of the environment was decreased, there was a reduction in SSBs at UHDR owing to the intertrack effect.

**Figure 3.**
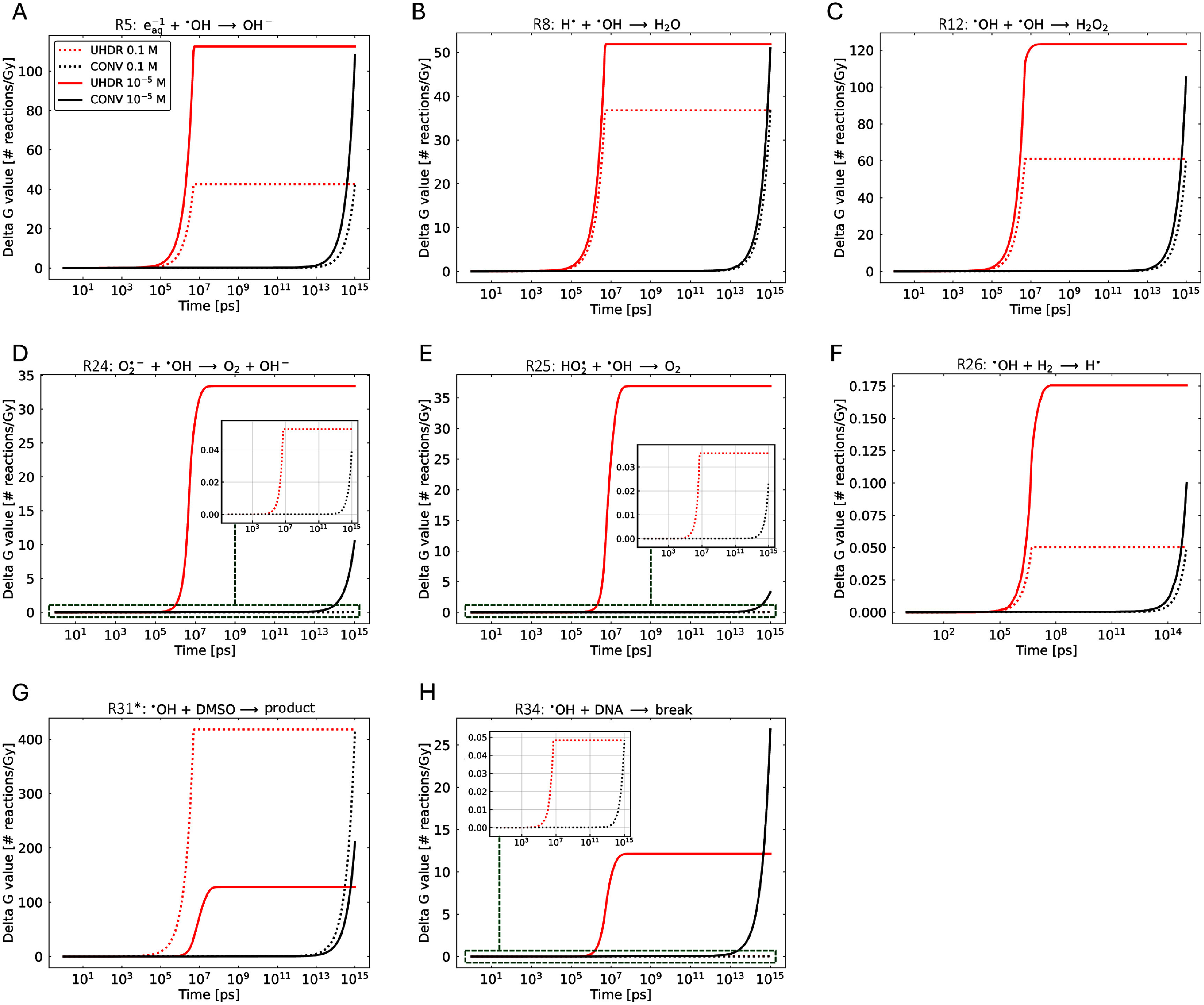
Computed delta G values as a function of time for the ${} _{\mathrm{n}}^\cdot{\mathrm{OH}}$ radical consumption reactions R5 (A), R8 (B), R12 (C), R24 (D), R25 (E), R26 (F), R31* (G), and R34 (H). UHDR results are shown in red and CONV results are shown in black. Simulation results for 0.1 M of DMSO (highest scavenging capacity) and 10^−5^ M of DMSO (lowest scavenging capacity) are depicted by the dotted and solid lines respectively. Zoomed insets were added to panels D, E, and H, since the delta G values for these reactions at 0.1 M of DMSO are approximately 3 orders of magnitude lower than at the higher scavenging capacity.

This intertrack effect is further explained by figure 3, which was created from the simulation outputs of the lowest (10^−5^ M DMSO) and highest (0.1 M DMSO) scavenging capacities at 21% O_2_ and for 100 Gy. Delta G values refer to the difference in species G values between successive MC steps. Consequently, the delta G values shown in each panel of figure 3 describes the cumulative number of times a specific reaction occurs at each time step in the simulation. At high doses within a radiation pulse, and at UHDR, the greater number of radiation tracks causes a large concentration of $e_{\mathrm{aq}}^ - $, ${\mathrm{O}}{{\mathrm{H}}^{\bullet }}$, and ${{\mathrm{H}}^{\bullet }}$ radicals. As shown in panels A, B, and C of figure 3, our model predicts that at low enough scavenging capacities the reduction in the yield of ${\mathrm{O}}{{\mathrm{H}}^{\bullet }}$ radicals, and hence its availability to induce DNA damage, is primarily due to an increase in the delta G of the second-order reactions R5, R8, and R12. Given the presence of oxygen, the large concentration of $e_{\mathrm{aq}}^ - $ and ${{\mathrm{H}}^{\bullet }}$ radicals also react with molecular oxygen through reactions R27–R30* of table 1, resulting in ${\mathrm{O}}_2^{ \bullet - }$ and ${\mathrm{HO}}_{\mathrm{2}}^{\bullet }$ anion radicals which themselves consume ${\mathrm{O}}{{\mathrm{H}}^{\bullet }}$ radicals through R24 and R25 respectively. Given its low reaction rate constant, the consumption of ${\mathrm{O}}{{\mathrm{H}}^{\bullet }}$ radicals through R26 is minor. These additional oxygen dependent ${\mathrm{O}}{{\mathrm{H}}^{\bullet }}$ consumption reactions are shown in panels D, E, and F of figure 3. At 10^−5^ M of DMSO, the scavenging capacity of the system is low enough that ${\mathrm{O}}{{\mathrm{H}}^{\bullet }}$ consumption by DMSO (panel G) competes with the reactions in panels A–E, thereby making the system more susceptible to dose-rate effects. Increased ${\mathrm{O}}{{\mathrm{H}}^{\bullet }}$ consumption by the reactions in panels A–E at UHDR therefore cause a decrease in the availability of ${\mathrm{O}}{{\mathrm{H}}^{\bullet }}$ radicals for stand break induction, shown in panel H. At the highest scavenging capacity, panel G depicts the dominance of ${\mathrm{O}}{{\mathrm{H}}^{\bullet }}$ radical consumption by DMSO, resulting in none of the other reactions being able to compete, thereby causing no statistically significant difference between the induced stand breaks at CONV and UHDR.

**Figure 4.**
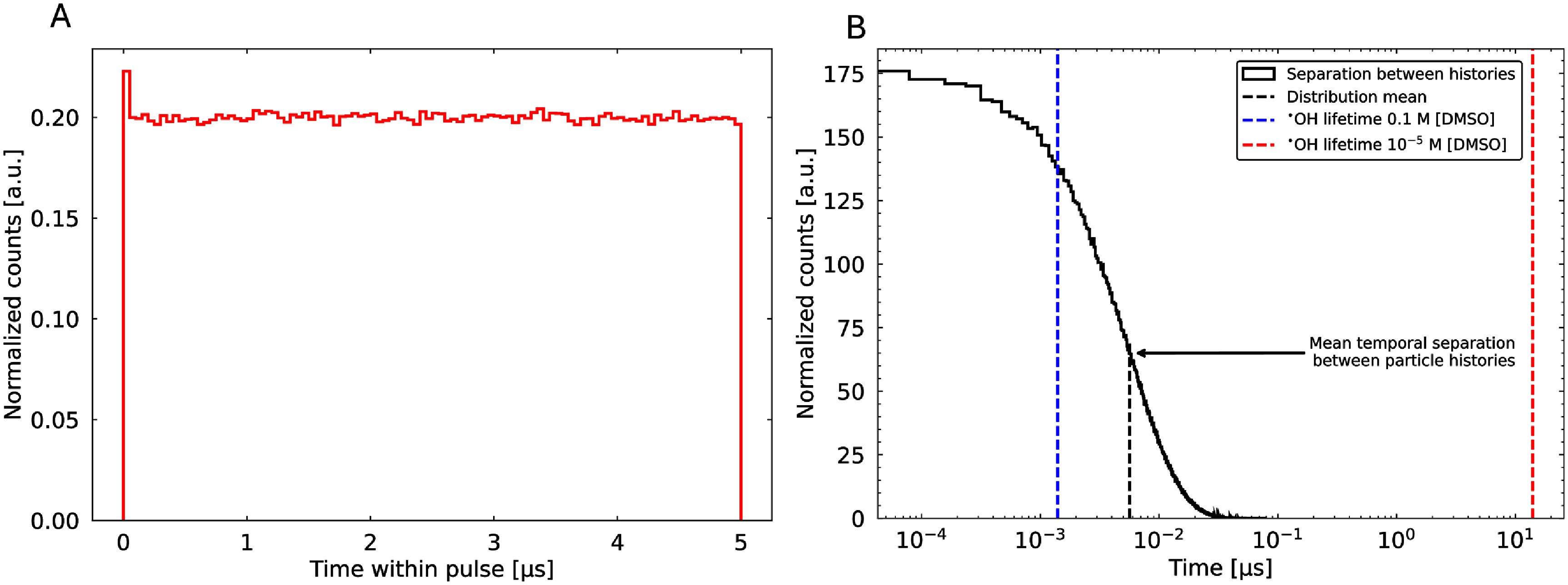
A single pulse of radiation delivered using TOPAS-nBio is depicted by the histogram in panel A. As described in section 2.2.1, the time assigned to each particle history within this distribution was sampled from a uniform distribution. The separation time between particle histories is depicted by the black distribution in panel B, in which it is compared to the mean ${} _{\text{ }}^{\bullet }{\mathrm{OH}}$ radical lifetimes at 0.1 M of DMSO (blue dashed line), and at 10^−5^ M of DMSO (red dashed line).

The intertrack effect at low scavenging capacities is qualitatively supported by panels A and B in figure 4. Panel A depicts a histogram of times associated to each particle history that makes up the uniform UHDR pulse of radiation that was delivered. The slight increase observed at the start is due to the first history of each independent run being automatically set to 1 ps. The distribution in panel B depicts how separated in time each successive particle history is, with a mean separation of ∼5.6 ns. The mean ${} _{\text{ }}^{\bullet }{\mathrm{OH}}$ radical lifetimes of ∼1.4 ns and ∼14 *μ*s correspond to 0.1 M and 10^−5^ M of DMSO respectively.

This figure qualitatively shows that at biologically relevant scavenging capacities (0.1 M), the average lifetime of ${} _{\text{ }}^{\bullet }{\mathrm{OH}}$ radical (∼1.4 ns) in the scavenging medium is shorter than the average temporal separation between particle tracks (∼5.6 ns), making intertrack interactions unlikely at a dose rate of 2 $ \times $ 10^7^ Gy s^−1^. For an intertrack interaction to occur at these scavenging capacities, both histories would need to be located in the part of the distribution to the left of the dashed blue line in panel E. In contrast, at low scavenging capacities (red dashed line), the average lifetime of an ${} _{\text{ }}^{\bullet }{\mathrm{OH}}$ radical (∼14 *μ*s) is long enough that any two histories within the distribution of particle tracks have the potential to undergo an intertrack interaction.

### 3.2. Double strand breaks

**Figure 5.**
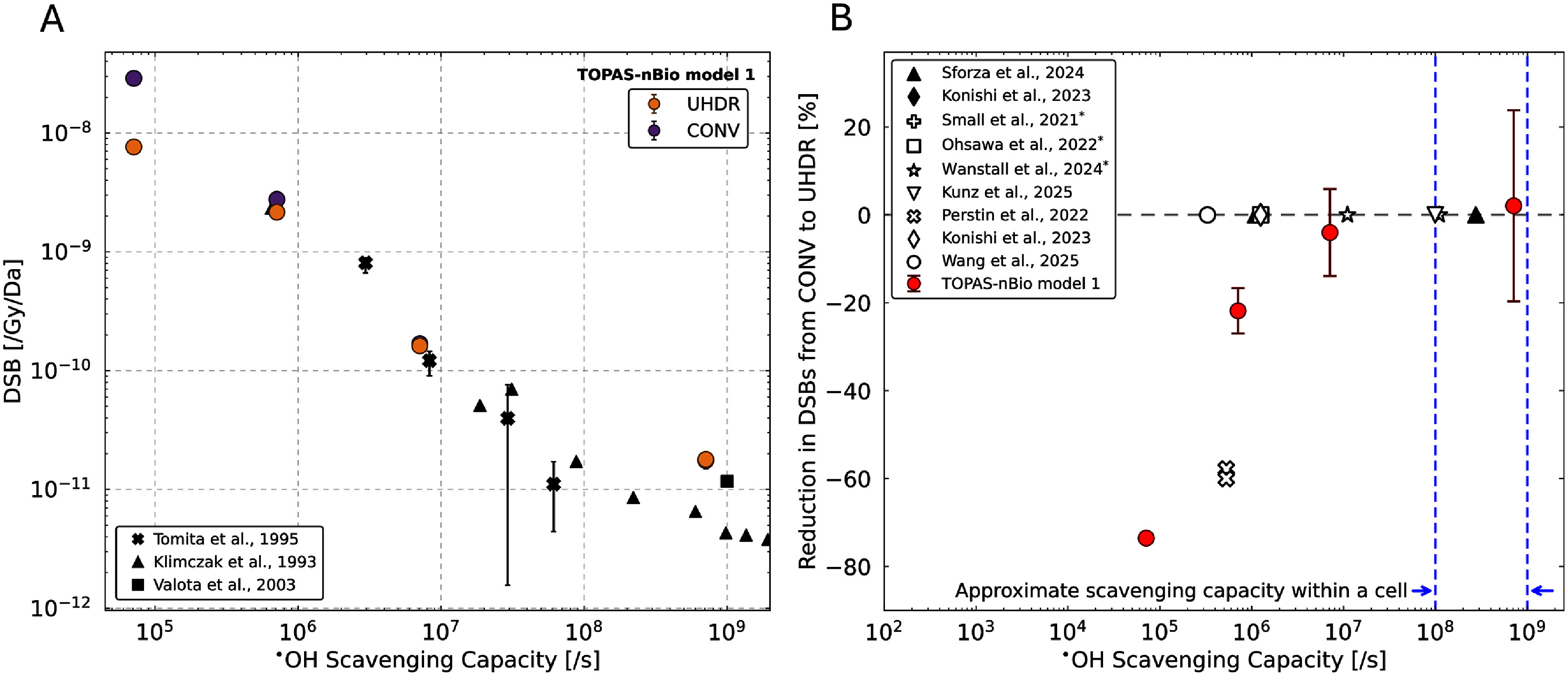
Panel (A) depicts the computed DSBs from model 1 as a function of ${} _{\text{ }}^{\bullet }{\mathrm{OH}}$ scavenging capacity against experimental (Klimczak *et al* 1993, Tomita *et al* 1995) and MC (Valota *et al* 2003) data. These values plotted in panel (A) are means, and error bars (not visible) correspond to standard deviations of the respective DSB distribution. Panel (B) depicts the percent reduction in DSBs from CONV to UHDR with propagated errors compared to experimental data (Small *et al* 2021, Ohsawa *et al* 2022, Perstin *et al* 2022, Konishi *et al* 2023, Sforza *et al* 2024, Wanstall *et al* 2024, Kunz *et al* 2025, Wang *et al* 2025).

Figure 5 depicts the DSBs obtained from model 1 as a function of ${} _{\text{ }}^{\bullet }{\mathrm{OH}}$ radical scavenging capacity. Distribution means are plotted in panel A, and error bars (smaller than the symbols, thus not visible) are representative of the corresponding distribution’s standard deviation. CONV DSBs (/Gy Da^−1^) from the lowest to highest scavenging capacity were (2.88 $ \pm $ 0.04) $ \times $ 10^−8^, (2.76 $ \pm $ 0.01) $ \times $ 10^−9^, (1.68 $ \pm $ 0.01) $ \times $ 10^−10^, and (1.76 $ \pm $ 0.26) $ \times $ 10^−11^, while for UHDR (/Gy Da^−1^) these values were (7.64 $ \pm $ 0.20) $ \times $ 10^−9^, (2.16 $ \pm $ 0.11) $ \times $ 10^−9^, (1.62 $ \pm $ 0.15) $ \times $ 10^−9^, and (1.79 $ \pm $ 0.27) $ \times $ 10^−11^. As depicted in panel A, the model for CONV agrees well with historical data at the low ${} _{\text{ }}^{\bullet }{\mathrm{OH}}$ radical scavenging capacities (Klimczak *et al* 1993, Tomita *et al* 1995) but overestimates the yields from Klimczak *et al* at the highest scavenging capacity. Nevertheless, the predicted value at the highest scavenging capacity is similar to the value obtained by PARTRAC (Valota *et al* 2003) for linear DNA, and is consistent with reported DSB induction yields on the order of 10^−11^ or 10^−12^ Gy Da^−1^ for cellular environments (Tomita *et al* 1995). This ‘leveling off’ at the highest scavenging capacity is a known phenomenon and is elaborated upon in the discussion.

Panel B of figure 5 depicts the percent reduction in DSBs as a function of scavenging capacity. Similarly to the SSBs, while there are differences in the mean DSBs induced at UHDR vs CONV at the lowest scavenging capacity (∼73.5%), there were no statistically significant differences observed at the two highest scavenging capacities. This result is in line with the majority of the experimental data that found no significant difference between UHDR and CONV in aerated conditions for similar absorbed doses.

**Figure 6.**
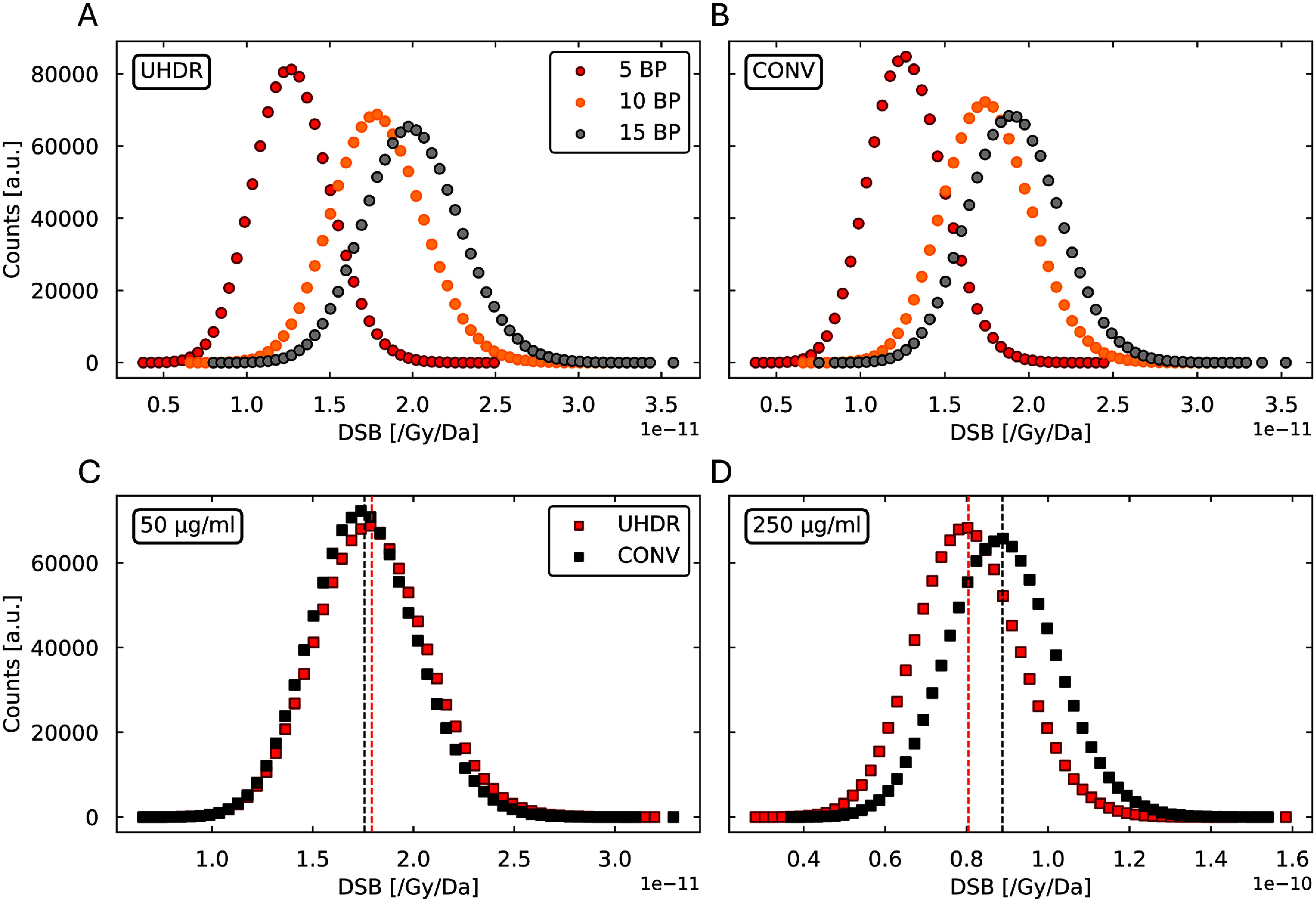
Distribution of DSBs at the highest scavenging capacity (0.1 M of DMSO) and results of the sensitivity analysis. The original conditions of model 1 were a 10 bp separation for DSBs, with a 50 *μ*g ml^−1^ DNA concentration. The corresponding distribution of DSBs is depicted in orange in panels (A) and (B) and represents the distribution from which the means and standard deviations of figure 5 were obtained. Sensitivity analysis results for different DSB bp distance thresholds is shown in the top row in red (5 bp) and gray (15 bp). Panels (C) and (D) depict the distribution of DSBs at CONV and UHDR for a 10 bp distance for 50 *μ*g ml^−1^ (C) and 250 *μ*g ml^−1^ (D). The dashed lines in red and black represent the mean of the UHDR and CONV distributions respectively. In all panels, a fixed bin width of 0.1 × 10^−13^ Gy Da^−1^ was chosen and bars were replaced by a single point at the corresponding bar’s midpoint.

The results of the sensitivity analysis are shown in figure 6. As depicted in panels A and B, the impact of reducing the bp distance required for a DSB to occur from 10 bp to 5 bp causes the mean DSB yield to drop from 1.79 to 1.28 ($ \times $10^−11^ Gy Da^−1^) for UHDR and from 1.76 to 1.28 ($ \times $10^−11^ Gy Da^−1^) for CONV. Conversely, increasing the distance threshold to 15 bp raises the mean DSBs to 2.00 and 1.92 ($ \times $10^−11^ Gy Da^−1^) for UHDR and CONV respectively. This behavior is expected. A stricter requirement (5 bp) reduces the number of SSB pairs which satisfy the conditions for a DSB to occur, causing the mean to drop. The converse holds true for relaxing the restriction to 15 bp. This interpretation is also consistent with the change in amplitude of the distributions, which is attributed primarily to the analysis methodology employed. For a fixed total number of repetitions (10^6^), the expected narrower distributions resulting from stricter DSB formation requirements necessarily have higher amplitudes, while the broader distributions have lower amplitudes. Standard deviations at 5, 10, and 15 bp were 2.30, 2.74, and 2.90 ($ \times $10^−12^ Gy Da^−1^) at UHDR, and 2.22, 2.60, and 2.74 ($ \times $10^−12^ Gy Da^−1^) at CONV.

Shown in panels C and D of figure 6 are the results of sensitivity analysis in which the DNA concentration was varied. At 50 *μ*g ml^−1^, the distributions are very similar, with small differences attributed to statistical fluctuations. Mean DSBs of 1.79 $ \times $ 10^−11^ Gy Da^−1^ and 1.76 $ \times $ 10^−11^ Gy Da^−1^ for UHDR and CONV respectively differ by <2%. Increasing the concentration of DNA by a factor of 5 (to 250 *μ*g ml^−1^) causes the mean DSB yields to increase to 8.04 $ \times $ 10^−11^ Gy Da^−1^ for UHDR, representing a factor of 4.5 increase compared to the 50 ug ml^−1^ concentration, and 8.88 $ \times $ 10^−11^ Gy Da^−1^ for CONV, representing a factor of 5 increase. At the highest DNA concentration, there was a 9.46% drop in mean DSBs from CONV to UHDR. For dilute plasmid solutions in which the volume is predominantly composed of water, an approximately linear increase of DSB yields with DNA concentration is expected.

## 4. Discussion

The role of differential DNA damage and repair as a determinant in the FLASH effect remains debated, with some reviews supporting this hypothesis (Friedl *et al* 2022), while others suggest that the hypothesis is implausible and does not play a fundamental role (Limoli and Vozenin 2023). Starting from the simpler plasmid systems, we used TOPAS-nBio to investigate potential differences in DNA damage induction under aerated conditions. The simulations depicted a reduction in SSBs when irradiating with a single pulse at UHDR (2 $ \times $ 10^7^ Gy s^−1^) compared to CONV (0.1 Gy s^−1^) at low ${} _{\text{ }}^{\bullet }{\mathrm{OH}}$ radical scavenging capacities (figure 2). This reduction comes about due to the intertrack effect and increased radical-radical reactions of ${} _{\text{ }}^{\bullet }{\mathrm{OH}}$ radicals with the products of oxygen consumption reactions R27–R30*, thereby reducing the number of ${} _{\text{ }}^{\bullet }{\mathrm{OH}}$ radicals available to induce indirect DNA damage (figure 3).

The trend of increased sparing by UHDR at low scavenging capacities was consistent with experimental observations. For example, Sforza *et al* and Wanstall *et al* both showed that all other conditions being the same, reducing the ${} _{\text{ }}^{\bullet }{\mathrm{OH}}$ radical scavenging capacity should result in a larger differential between UHDR and CONV induced SSBs (2024, 2024). The results of this work suggest that the reduction in DNA damage observed from experiments performed at the low ${} _{\text{ }}^{\bullet }{\mathrm{OH}}$ radical scavenging capacities (Ohsawa *et al* 2022, Perstin *et al* 2022, Konishi *et al* 2023, Sforza *et al* 2024, Wang *et al* 2025) could partially be due to the intertrack effect. While physico-chemically induced differences in DNA damage between UHDR and CONV can be studied with plasmid assays, correlating these findings with the FLASH effect, which is a biological effect, requires biologically relevant scavenging capacities within the plasmid solution. Under these conditions, the spatio-temporal distribution of induced breaks become biologically more relevant, and model 1 predicts that the intertrack effect is lost at intracellular scavenging capacities. The work of Sforza *et al* similarly shows that at biologically relevant scavenging levels, under aerated conditions, there is no significant difference in strand break yields (Sforza *et al* 2024). However, Given the five orders of magnitude difference in dose rates used (2 $ \times $ 10^7^ in this work, compared to 125 Gy s^−1^ in Sforza *et al*), the reduction in SSB yields observed by Sforza *et al* at low scavenging capacities may not be due to intertrack reactions, as the authors point out.

Model 2 simulations included WR-1065, which introduced a mechanism of oxygen competition at the biologically relevant scavenging capacity, leading to a more *in vivo*-like environment. Induced differences in DNA damage between UHDR and CONV in this environment could shed light on the underlying chemical reaction pathways that may be playing a role in the reduction in DNA damage at UHDR as observed in the *in vivo* studies. Despite its inclusion, we observed no significant differences between UHDR and CONV induced SSBs at biologically relevant scavenging capacities. This work used 1 mM of WR-1065 given that this concentration of WR-1065 at 21% O_2_ was shown to match expected hypoxia reduction factor values (D-Kondo *et al* 2024). However, D-Kondo *et al* made use of a more detailed DNA model, in which the 5 hydrogen abstraction sites of the DNA molecule were considered. Consequently, higher concentrations of WR-1065 inducing greater rates of chemical repair may be more appropriate for the simplistic DNA reaction models evaluated in this work. Future work should explore a range of WR-1065 concentrations while accounting for their impact on ${} _{\text{ }}^{\bullet }{\mathrm{OH}}$ radical scavenging, as well as variations in oxygen and DNA concentrations. In order for the system to remain at biologically relevant scavenging capacities, the concentration of any other scavenger within the system would need to be modulated.

There is also uncertainty in the rate constants used. Milligan *et al* calculated a rate constant for SSB repair by WR-1065 (R43*) of approximately 9.4 10^7^ M s^−1^ at 0.1 M of DMSO. This, however, was calculated for an O_2_ concentration of 0.25 mM and with the assumption that the oxygen fixation rate constant (R40/R41*) was 4.0 $ \times $ 10^8^ /M/s (Michaels and Hunt 1977, Milligan *et al* 1995). While the different O_2_ concentration used in this work (0.27 mM) is unlikely to result in meaningful differences, other literature references 2.0 $ \times $ 10^9^ M s^−1^ as the rate constant for R40/R41* (Von Sonntag 2006), which is 3 times higher than the value used for model 2. Despite these limitations/uncertainties, model 2 predicts no differences in chemical repair between UHDR and CONV at biologically relevant scavenging capacities, providing further arguments in favor of the conclusions drawn from *in vitro* work (Barghouth *et al* 2023).

Ratios of CONV to UHDR induced damages calculated from SSB and DSB induction rates (Small *et al* 2021, Ohsawa *et al* 2022, Wanstall *et al* 2024) were included in figures 2 and 5. In contrast to the ratios calculated from raw plasmid fractions, using the induction rates may not be ideal to characterize the underlying effects as these rates average any potential differences over the entire range of doses studied. This is significant as the largest differences between UHDR and CONV induced plasmid DNA damage is often observed at the highest doses studied. Consequently, these ratios calculated from SSB/DSB induction rates may have additional uncertainties linked with this averaging effect, as well as differences between the models used to calculate these rates (Cowan *et al* 1987, McMahon and Currell 2011).

Experimental studies in which enzymes were used to convert base damages to strand breaks were also included in figures 2 and 5. As observed by Konishi *et al*, the introduction of these enzymes resulted in an increase in the DSB and SSB yields for both CONV and UHDR as expected (Konishi *et al* 2023). Interestingly, the reduction in SSBs from CONV to UHDR is smaller when Fpg is present compared to its absence. This suggests a differential induction of base damages depending on the dose rate. These base damages are not explicitly modeled in TOPAS-nBio. While the computational plasmid geometry does have structures corresponding to DNA bases, and while the rate constants for ${} _{\text{ }}^{\bullet }{\mathrm{OH}}$, $e_{\mathrm{aq}}^ - $, and ${{\mathrm{H}}^{\bullet }}$ reactions with DNA bases exist in literature (Buxton *et al* 1988), at these small scales the reaction rate constant between a reactive pair may be influenced by the presence of its neighbors (Bluett and Green 2006). These effects are further discussed elsewhere (Ramos-Méndez *et al* 2021, Tran *et al* 2021). Nevertheless, the authors note that use of the IRT while disregarding these variable rates may still be well-suited for the modeling of DNA damage with nucleotide/base-pair level resolution.

As shown in figure 5, model 1 predicts no significant differences in the mean DSBs induced between UHDR and CONV at biologically relevant scavenging capacities and aerated conditions. When plotting strand breaks as a function of ${} _{\text{ }}^{\bullet }{\mathrm{OH}}$ radical scavenging capacity on a log-log scale, an approximately linear trend is expected over intermediate scavenging capacities. However, as shown in panel A, the yields deviate from linearity at high scavenging capacities, instead exhibiting a shallow concave curvature with mean DSB yields higher than those predicted by a linear trend line. This non-linearity at scavenging capacities >10^8^ /s is a known phenomenon (Krisch *et al* 1991, Klimczak *et al* 1993, Milligan *et al* 1993) and is attributed to a combination of direct DNA damage and locally multiply damaged sites (Ward 1985). Under these conditions, only those ${} _{\text{ }}^{\bullet }{\mathrm{OH}}$ radicals generated in close proximity to a DNA molecule induce indirect strand breaks. As shown in panel B of figure 5, the majority of the experimental data points report no statistically significant differences in DSBs. These experimental results are effectively consistent with the model 1 DSB results at scavenging capacities of 0.001 M, and 0.1 M, however the decision was made to plot the actual ratios for model 1 with error bars instead of simply placing them at 0 on the *y*-axis. Perstin *et al* is the only study in which statistically significant differences in DSBs was observed (at 30 Gy), however it should be noted that at slightly lower doses the DSBs induced by CONV and UHDR are also no longer significantly different (Perstin *et al* 2022). Furthermore, the authors observed linear DNA isoform yields of only a few percent at most. This is in line with other studies that reported linear band intensities close to the detection limit of agarose gel electrophoresis, resulting in non-significant differences in DSBs between UHDR and CONV and inhibiting further comparative analysis (Kacem *et al* 2022, Sforza *et al* 2024, Wanstall *et al* 2024). This is further motivation to use techniques such as atomic force microscopy to investigate plasmid strand breaks, which may also provide more information about the complexity of DNA damage (Pang *et al* 2015).

The sensitivity analysis performed at the highest scavenging capacity revealed an expected decrease in mean DSBs when the threshold distance for DSB induction was decreased. Relative to the 10 bp threshold we observed a 29% reduction and 12% increase in DSB yields for the 5 and 10 bp thresholds respectively at UHDR, and a 27% reduction and 9% increase for CONV. These differences, arising from the sparsely ionizing low LET x-rays, are larger than what would be expected of higher LET radiation, which produces more densely clustered ionizations. The use of 10 bp in this work is consistent with Tomita *et al.*, who observed good agreement between MC calculated DSB yields and experimental measurements when a 10 bp threshold was used. Furthermore, their DSB yields similarly decreased when the threshold was changed from 15, down to 10 or 5 bp (Tomita *et al* 1998).

Increasing the DNA concentration from 50 *μ*g ml^−1^ to 250 *μ*g ml^−1^ resulted in a directly proportional factor of 5 increase in the mean CONV DSB yields. This is to be expected for dilute plasmid solutions. Interestingly, there was a lower increase in mean UHDR DSB yields (factor of 4.5), resulting in 9.46% less DSBs at UHDR compared to CONV at the higher DNA concentration. This non-linearity has been observed previously and results in a plateau at high DNA concentrations when plotting strand breaks as a function of DNA concentration (Milligan *et al* 1993, Milligan and Ward 1994). However, in the work of Milligan *et al*, this deviation from linearity and plateauing of strand breaks only occurs above 1000 *μ*g ml^−1^ of DNA. We hypothesize that significantly higher DNA concentrations may make the system more sensitive to intertrack effects, reducing the number of ${} _{\text{ }}^{\bullet }{\mathrm{OH}}$ radicals able to induce damage, and thereby resulting in a 9.46% decrease in the mean DSBs at UHDR compared to CONV. In dilute plasmid solutions and at low scavenging capacities, the intertrack effect manifests as a reduction in SSBs at UHDR. This effect is lost when the bulk scavenging capacity of the system, induced by DMSO, becomes comparable to the biological scavenging capacity within a cell. However, by significantly increasing the concentration of DNA at these biological scavenging capacities, a greater proportion of ${} _{\text{ }}^{\bullet }{\mathrm{OH}}$ radicals surviving this bulk scavenging interact with DNA. These radicals are necessarily short lived, and therefore any process (i.e. intertrack effects) that modify the yield of such radicals at early times may become more apparent. This hypothesis only applies to indirect DNA damage, and no difference between UHDR and CONV would therefore be expected for the direct DNA damage component. Nevertheless, a modest 8% decrease in direct DNA damage at UHDR was also observed. Under these irradiation conditions, however, 91% of the total DNA damage was indirect. As a result, the apparent reduction in direct damage should be interpreted with caution, and a more rigorous assessment would require a simulation framework specifically optimized for studying direct damage, such as using higher-LET particles.

One limitation of the TOPAS-nBio models presented in this work is the static O_2_ concentration during irradiation, and the lack of reactions with downstream scavenger-derived secondary radicals. All simulations were performed in an environment where oxygen is abundant (21%), however there is interest in looking at low oxygen environments as *ex vivo* (Cooper *et al* 2022) and *in vitro* (Tessonnier *et al* 2021) studies respectively demonstrated that a reduction in DNA damage at UHDR was only obtained at oxygenation levels ⩽0.5% or ⩽ 1%. Simulating a static 1% O_2_ is not appropriate, as irradiations at this level of oxygenation could completely deplete the oxygen. This effect would not be captured by the current model since it would require sets of reactions in table 1 to no longer contribute to the chemical kinetics of the system. Nevertheless, under hypoxic conditions no differences in plasmid DNA damage were observed experimentally (Sforza *et al* 2024, Kunz *et al* 2025), perhaps linked to the lack of repair processes. These considerations for oxygen would also have an impact on the modeling of scavenger-derived secondary radicals. In the case of DMSO, the methyl radical has been shown to contribute to the yield of SSBs, particularly in the absence of oxygen (Milligan and Ward 1994). These downstream radicals may be playing a role in the work of Kunz *et al*, who used DMSO as their radical scavenger (2025). Other studies irradiated plasmids in solutions containing residual EDTA, which also results in the creation of EDTA-derived secondary radicals (Höbel and Sonntag 1998). TRIS may be the more appropriate ${} _{\text{ }}^{\bullet }{\mathrm{OH}}$ radical scavenger to use in these kinds of studies given that it is unreactive with biomolecules such as DNA (Krisch *et al* 1991). Additionally, radiation induced oxidation of WR-1065 can undergo further chain reactions in the presence of oxygen (Becker *et al* 1994, Milligan *et al* 1995). Lastly, while this work characterized differences in plasmid DNA damage between UHDR and CONV depending on the scavenging capacity of the system, these models are not sufficient to draw conclusions about differential responses in normal and tumor tissues, where additional factors such as a dynamic oxygen concentration, pH, and vasculature would need to be considered. Implementation of appropriate models to address these limitations are avenues of future research.

## 5. Conclusion

In this work we used the MC code TOPAS-nBio to investigate differences in pUC19 plasmid DNA damage irradiated at 100 Gy at UHDR and CONV for various radical scavenger concentrations at atmospheric oxygen conditions. At the lowest scavenging capacity, the intertrack effect causes a reduction in both SSBs and DSBs when irradiating at UHDR. However, at intracellular scavenging capacities there were no statistically significant differences in DNA damage induction between UHDR and CONV, and the DNA damage resulting from the introduction of WR-1065 to mimic *in vivo* repair was not significantly different between the dose rates.

## Data Availability

All data that support the findings of this study are included within the article (and any supplementary information files).
